# Prevalence of Fowl Adenovirus Serotype 4 and Co-Infection by Immunosuppressive Viruses in Fowl with Hydropericardium Hepatitis Syndrome in Shandong Province, China

**DOI:** 10.3390/v11060517

**Published:** 2019-06-05

**Authors:** Guanliu Yu, Yun Lin, Yanguo Dou, Yi Tang, Youxiang Diao

**Affiliations:** 1 College of Animal Science and Technology, Shandong Agricultural University, 61 Daizong Road, Tai’an 271018, Shandong Province, China; yuguanliu@163.com (G.Y.); lyun1994@126.com (Y.L.); yangg_dou@163.com (Y.D.); 2 Shandong Provincial Key Laboratory of Animal Biotechnology and Disease Control and Prevention, Shandong Agricultural University, 61 Daizong Road, Tai’an 271018, Shandong Province, China; 3 Shandong Provincial Engineering Technology Research Center of Animal Disease Control and Prevention, Shandong Agricultural University, 61 Daizong Road, Tai’an 271018, Shandong Province, China

**Keywords:** poultry, fowl adenovirus serotype 4, hydropericardium hepatitis syndrome, epidemiological investigation, immunosuppressive viruses

## Abstract

Fowl adenovirus serotype 4 (FAdV-4) is the pathogenic agent of hydropericardium hepatitis syndrome (HHS) in chickens and ducks, which has caused huge economic losses for the Chinese poultry industry since 2015. In order to objectively determine the prevalence and co-infection status of the virus in Shandong province in China, we analyzed a total of 679 clinical cases of chickens and ducks from 36 farms in the province. The results showed that the FAdV-4 infection rate was 65.2% (443/679), and the rate in breeder ducks was almost two-fold higher than that in breeder chickens (68.57% vs. 34.30%). Notably, co-infection by H9N2 avian influenza virus, infectious bursal disease virus, and/or chicken infectious anemia virus was very common in the 443 FAdV-4-positive cases. Furthermore, phylogenetic analysis of the hexon genes of four Shandong FAdV-4 isolates revealed that these strains clustered into Indian reference strains, indicating that the Shandong FAdV-4 strains might have originated in India. These findings provide the first data on the prevalence and co-infection status of FAdV-4 in Shandong province, which may serve as a foundation for the prevention of FAdV-4 in the field.

## 1. Introduction

Fowl adenoviruses (FAdVs) are members of the *Aviadenovirus* genus, *Adenoviridae* family. As such, they contain linear, double-stranded DNA. The viruses are divided into five species (FAdV-A to FAdV-E) and 12 serotypes (FAdV-1 to FAdV-8a and FAdV-8b to FAdV-11) [1,2]. Different serotypes of FAdVs can cause different clinical signs in poultry flocks. For example, FAdV-1 can induce gizzard erosion in chickens [3,4,5,6,7,8,9,10,11], inclusion body hepatitis (IBH) in chickens is often associated with FAdV-2, -8a, -8b, or -11 [12], and so on. Notably, FAdV-4 is the causative agent of hydropericardium hepatitis syndrome (HHS) and IBH in chickens and ducks [13,14], and its diseases have caused severe economic losses in the global poultry industry for more than 30 years [15].

In China, there have been a large number of FAdV-4-mediated HHS outbreaks in chickens and ducks since 2015 [16,17]. In Shandong province, one of the largest poultry breeding areas in China, these fowl are commonly affected by various immunosuppressive viruses, including H9N2 avian influenza virus (AIV), FAdV-4, infectious bursal disease virus (IBDV), and chicken infectious anemia virus (CIAV). In addition, there are also reports of viral co-infection, such as by duck circovirus (DuCV) and duck hepatitis virus 1 (DHV-1) [18]; H9N2 AIV and Tembusu virus (TMUV) [19]; Marek’s disease virus (MDV) and CIAV [20]; and duck hepatitis A virus (DHAV-1) and DHAV-3 [21]. Currently, although FAdV-4 can clearly cause HHS on its own in chickens and ducks [22,23,24,25], both the extent of the FAdV-4 infection and degree of co-infection in the area remain unclear. Therefore, to better understand the prevalence of FAdV-4 and the incidence of co-infection by immunosuppressive viruses, an epidemiological survey of FAdV-4 infection in chickens and ducks with HHS in the province was conducted.

## 2. Materials and Methods 

### 2.1. Sample Collection and Treatment

In this study, a total of 679 clinical cases of ducks and chickens were collected from 36 suspected FAdV-4-infected farms in nine cities in Shandong province between June 2015 and December 2016. This study was approved by the Animal Care and Use Committee of the Shandong Agricultural University (permit number: 2016093, March, 2016). All the experimental animals of this study were cared for and maintained throughout the experiments by strictly following the Guidelines of Experimental Animals of the Ministry of Science and Technology (Beijing, China).

Oral or cloacal swab samples from each case were homogenized, diluted 1:10 in phosphate-buffered saline containing mycillin (20% *w/v*) and centrifuged at 6000× *g* for 20 min at 4 °C. The supernatant was then filtered through a 0.22-μm syringe-driven filter (Thermo Scientific, Lenexa, KS, USA) for DNA or RNA extraction. As for the virus isolation, the filtered suspension was then inoculated into 9-day-old specific-pathogen-free (SPF) chicken embryos (0.2 mL/embryo) through yolk sac route. After four passages in embryonated eggs, embryo death occurred on passage days 3–5. The allantoic fluids were harvested in biohazard safety equipment as viral stocks and stored at −80 °C for further study.

### 2.2. Detection of FAdV-4, H9N2 AIV, IBDV, and CIAV

Total viral DNA was extracted from half of each supernatant sample using a DNeasy Tissue Kit (Qiagen, Hilden, Germany) for the detection of FAdV-4 and CIAV. Total viral RNA was extracted from the other half of each sample using Trizol (TaKaRa, Dalian, China) for the detection of H9N2 AIV and IBDV according to the manufacturer’s instructions. The extracted RNA was then used for cDNA synthesis using TransScript^®^ All-in-One First-Strand cDNA Synthesis SuperMix (TransGen Biotech, Beijing, China). The primers used for FAdV-4, H9N2 AIV, IBDV, and CIAV detection were originally designed according to conserved sequences of the above strains in GenBank (Table 1).

The synthesized genes were amplified from the cDNA by PCR utilizing Cwbio EsTaq MasterMix (Cwbio, Shanghai, China) with the following parameters: 94 °C for 4 min followed by 30 cycles of 94 °C for 40 s, 55 °C for 40 s, 72 °C for 45 s, and a final elongation step of 72 °C for 8 min. All PCR products were analyzed by electrophoresis on a 1.0% agarose gel.

### 2.3. Phylogenetic Analysis

In this study, the FAdV-4 strains SDLC, SDD01, SDJX, and SDSX were isolated from breeder chickens, commercial chickens, breeder ducks, and commercial ducks, respectively, and used as representative strains for phylogenetic analysis of FAdV-4 in Shandong province. The entire hexon gene open reading frames of the four strains were amplified by PCR using TransScript^®^ DNA Polymerase High Fidelity (HiFi; TransGen Biotech, Beijing, China) with two pairs of primers (Table 1) according to our previously described procedures [24]. 

All PCR products were visualized by electrophoresis in a 1.0% (*w/v*) agarose gel containing ethidium bromide and subsequently purified using a Gel Band Purification Kit, per the manufacturer’s instructions. The purified product was cloned into pMD18-T vectors (TaKaRa, Dalian, China) and sequenced using the Sanger method (Sangon Biotech, Shanghai, China).

ClustalW in the MegAlign program of DNAStar (Version 6.0, Madison, WI, USA) was used to align the amplified fragments of the hexon gene. Phylogenetic trees were then created based on the complete FAdV hexon gene. The trees included the four FAdV-4 isolates and 30 other reference strains and were developed in MEGA 5.0 (http://www.megasoftware.net/) using the maximum likelihood method, based on the Tamura–Nei model with 1000 replicates [26].

### 2.4. Data Analysis

In this study, the positive rate of infection was calculated as the percentage of the total tested samples testing positive for FAdV-4 nucleic acids. The rate of co-infection was calculated as the percentage of the total tested samples testing positive for more than one type of viral nucleic acid.

## 3. Results

### 3.1. Epidemiology of FAdV-4 in HHS

**Figure 1:**
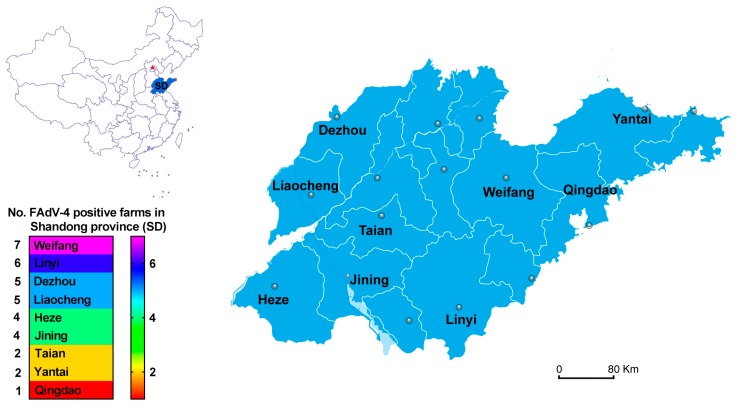
Geographical distribution of farms testing positive for FAdV-4 in Shandong province, China. Key cities within the province are represented by dots, and those cities from which positive samples were collected are named. The number of FAdV-4-positive farms involved in the study from each city were as follows: Dezhou (5), Heze (4), Jining (4), Liaocheng (5), Linyi (6), Qingdao (1), Taian (2), Weifang (7), and Yantai (2).

In this study, a total of 679 animal cases were collected from 36 suspected FAdV-4-infected farms in Shandong province. Of the 679 samples, 443 (65.24%) tested positive for FAdV-4 nucleic acid (Table 2). The spatial distribution is described in Figure 1. FAdV-4 has been circulating in more than half of the cities in Shandong province (nine of 16). 

### 3.2. FAdV-4 Co-Infection Rate

**Figure 2:**
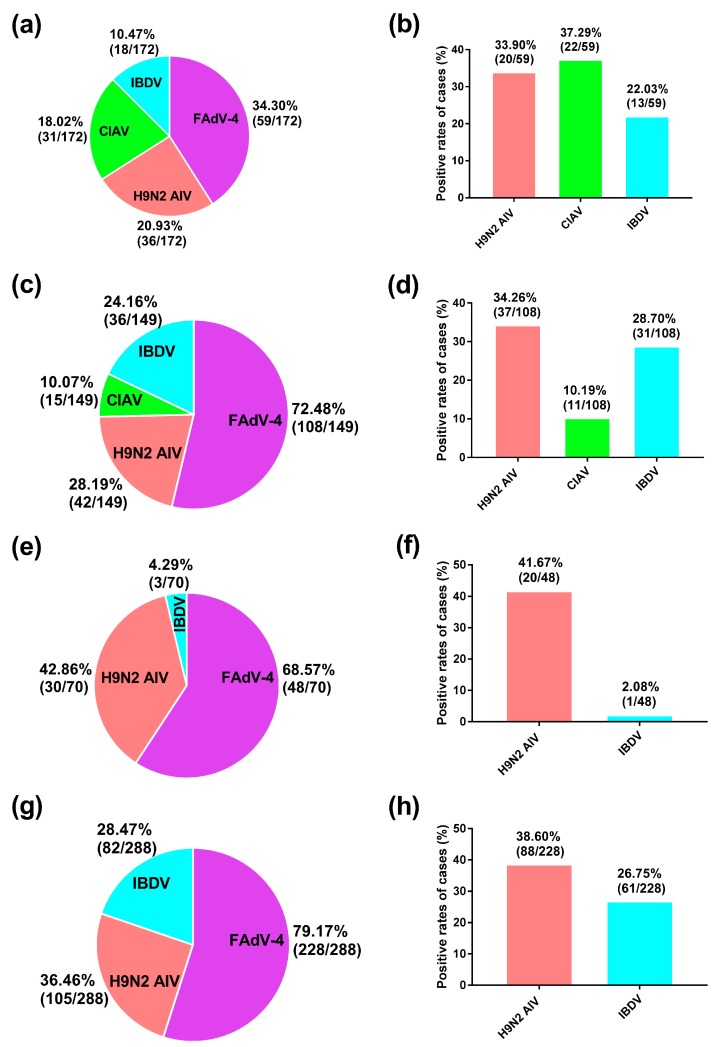
Co-infection by other viruses and FAdV-4 in tested samples. Note: (**a**,**c**,**e**,**g**) Pie charts showing the rates of infection of FAdV-4, H9N2 AIV, CIAV, and IBDV in the total population of the sampled fowl (*n* = 679) sub-grouped by species and farming purpose as breeder chickens (**a**), commercial chickens (**c**), breeder ducks (**e**), and commercial ducks (**g**). (**b**,**d**,**f**,**h**) Co-infection rates for the 443 FAdV-4-infected sample animals with H9N2 AIV, CIAV (chickens only), and IBDV. Abbreviations: CIAV: chicken infectious anemia virus; FAdV-4: fowl adenovirus serotype 4; H9N2 AIV: H9N2 avian influenza virus; IBDV: infectious bursal disease virus.

The rates of FAdV-4-positive samples in breeder chickens (Figure 2a), commercial chickens (Figure 2c), breeder ducks (Figure 2e), and commercial ducks (Figure 2g) were 34.30% (59/172), 72.48% (108/149), 68.57% (48/70), and 79.17% (228/288), respectively. Notably, breeder ducks were FAdV-4-positive almost twice as often as breeder chickens (68.57% vs. 34.30%, respectively).

We also determined the co-infection rates of FAdV-4 and one or more of three viral immunosuppressive diseases (i.e., H9N2 AIV, CIAV, IBDV) endemic to provincial farms, sub-grouped according to animal type. Because CIAV does not infect ducks, they were tested only for H9N2 AIV and IBDV. Among the 59 FAdV-4-positive breeder chicken samples (Figure 2b), the co-infection rates of H9N2 AIV, CIAV, and IBDV were 33.90% (20/59), 37.29% (22/59), and 22.03% (13/59), respectively. Among the 108 FAdV-4-positive commercial chicken samples (Figure 2d), the co-infection rates of H9N2 AIV, CIAV, and IBDV were 34.26% (37/108), 10.19% (11/108), and 28.70% (31/108), respectively. Among the 48 FAdV-4-positive breeder duck samples (Figure 2f), the co-infection rates of H9N2 AIV and IBDV were 41.67% (20/48) and 2.08% (1/48), respectively. Among the 228 FAdV-4-positive commercial duck samples (Figure 2h), the co-infection rates of H9N2 AIV and IBDV were 38.60% (88/228) and 26.75% (61/228), respectively (Figure 2h). The above results reveal that co-infection was widespread regardless of the animal type (i.e., species and farming purpose).

### 3.3. Sequencing and Phylogenetic Analysis

**Figure 3:**
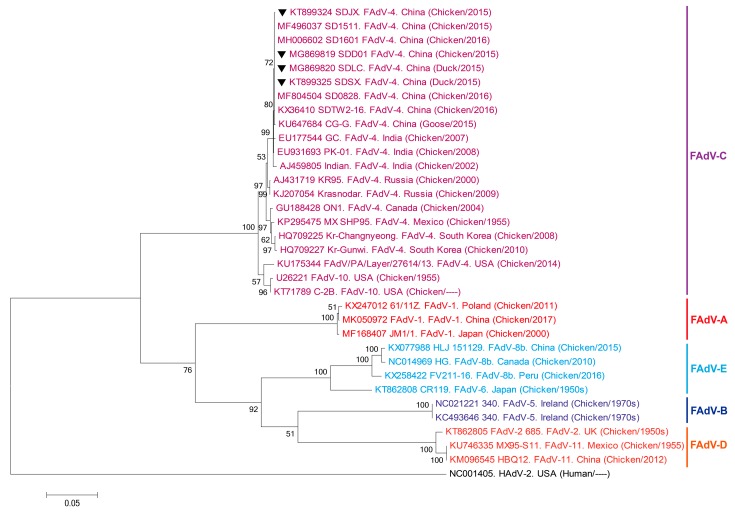
Phylogenetic tree of the hexon gene nucleotide sequences. Gene sequences of the SDJX, SDLC, SDSX, and SDD01 strains isolated from test samples and 30 other representative adenovirus strains constructed by the maximum likelihood method in MEGA 5.0. Bootstrap majority consensus values based on 1000 replicates are indicated at each branch point as a percentage. The location, source, and approximate dates of isolation are provided for each tree member. Black triangles indicate the four isolated Shandong strains. The GenBank numbers of reference strains are presented ahead of the reference strains. The scale bar indicates the number of nucleotide substitutions per site.

Phylogenetic analysis of the hexon gene sequences of the four representative FAdV-4 strains (i.e., SDJX, SDLC, SDSX, and SDD01) isolated in Shandong province (Figure 3) showed that they were mainly clustered in the FAdV-C group (e.g., GC and PK-01 strains), which were isolated in India.

## 4. Discussion

To the best of our knowledge, this is the first study providing epidemiological characteristics, including virus co-infection rates, of FAdV-4 in chickens and ducks in Shandong province, China. We found a rate of FAdV-4 infection of about 65% overall, though the rate in breeder chickens was lower than those in the other subgroups. Co-infection of Shandong province poultry with FAdV-4 and three (two for ducks) endemic immunosuppressive pathogens (i.e., H9N2 AIV, CIAV, and IBDV) was fairly common, especially for H9N2 AIV.

A better understanding of the epidemiological trends of infectious diseases in the field is critical for determining the proper measures needed to control their epidemics [19]. Here, the epidemiological data revealed that FAdV-4 had reached more than half of the key cities in Shandong province (9/16). We previously identified it in only six cities in December 2015 (unpublished data). Such findings indicate that the virus has expanded its geographic range, implying it may pose a threat to the health of poultry farms in surrounding cities and neighboring provinces. They also demonstrate the need for immediate attention to curtail the spread of the virus.

In recent years, the rapid development of the poultry breeding industry has caused growth primarily in FAdV, H9N2 AIV, CIAV, and IBDV infection. The epidemics of these pathogens are becoming increasingly severe, causing huge economic losses to the industry [27]. Notably, although H9N2 AIV is considered to have low pathogenicity for waterfowl, co-infection of the virus with other pathogens still poses a significant threat to waterfowl (and chickens) in the poultry industry, as well as human (public) health [19]. CIAV is an immunosuppressive pathogen that causes the hemorrhage of subcutaneous or muscle tissues, aplastic anemia, and thymic atrophy in chickens [27]. It is also a relatively common co-infective with FAdV [28]. IBDV is also an acute, highly contagious, and immunosuppressive fowl virus, which can cause high morbidity and mortality rates in susceptible birds [29].

In this study, the co-infection rates of FAdV-4 and the other immunosuppressive viruses were high among the tested fowl. Such a high infection rate by immunosuppressive pathogens can damage group immunity, providing favorable conditions for invasion by other immunosuppressive pathogens (e.g., IBDV, CIAV).

Interestingly, FAdV-4 in breeder ducks was almost twice as common as in breeder chickens. This may be due to the different growth habits of chickens and ducks. Ducks’ extensive time spent in water exposes them to waterborne contaminants far more than would be expected for chickens. This brings with it practical concerns, such as building healthy environments, improving breeding health management, and providing regular ventilation and disinfection [18,30].

Hexon, as a vital antigenic/structural protein of FAdV-4, has various antigenic determinants of genotype, species, and subspecies, and is often used to analyze the genetic evolutionary relationship of FAdV-4 [31,32,33]. Here, the phylogenetic analysis of the hexon gene of the four representative FAdV-4 strains isolated in Shandong province showed that they were mainly clustered in the FAdV-C group of India (e.g., GC, PK-01, and Indian strains). Therefore, we speculate that FAdV-4 infection in Shandong may have originated in birds from India.

In conclusion, this study describes the prevalence and co-infection characteristics of FAdV-4 in Shandong province for the first time, and thus may serve as a foundation for the prevention of FAdV-4 in the field.

## Figures and Tables

**Table 1:** Primers used in this study.

Primers	Sequence (5′–3′)	Size (bp)	Purpose
Hexon	F1: TGGACATGGGGGCGACCTA	1219	FAdV-4 hexon gene amplification
	R1: AAGGGATTGACGTTGTCCA		
	F2: AACGTCAATCCCTTCAACCACC	1350	
	R2: TTGCCTGTGGCGAAAGGCG		
FAdV-4	F: CTCTTCGACCTCGTGTCTTACA	568	FAdV-4 detection
	R: TTTACACGGCGTTGCCTGT		
H9N2 AIV	F: GATAGAGACTCAACCCAAAA	315	H9N2 AIV detection
	R: AACATCCTTTCCCATCTTCC		
IBDV	F: AGGCCCAGAGTCTACACCAT	475	IBDV detection
	R: CTGTTGCCACTCTTTCGTAGG		
CIAV	F: AATGAACGCTCTCCAAGAAG	582	CITV detection
	R: AGCGGATAGTCATAGTAGAT		

**Table 2:** FAdV-4 positive rates of clinical samples.

Fowl Species	No. of Cases	No. of FAdV-4 Positive Cases	% FAdV-4 Positives
Breeder chickens	172	59	34.30%
Commercial chickens	149	108	72.48%
Breeder ducks	70	48	68.57%
Commercial ducks	288	228	79.17%
Total	679	443	65.24%
